# Zidesamtinib in ROS1-positive NSCLC with brain metastases: clinical evidence and therapeutic implications

**DOI:** 10.1097/MS9.0000000000005710

**Published:** 2026-09-09

**Authors:** Abdihakim Sheikh, Saima Saleem, Sakan Binte Imran, Ehtisham Haider

**Affiliations:** a Queensland health, Rockhampton City, QLD, Pakistan; b Jinnah University for Women, Karachi, Pakistan; c FCPS Part 2 Trainee, Cardiology, Sir Salimullah Medical College, Mitford Hospital, Dhaka, Bangladesh; d FCPS Part 2 Trainee, Nephrology, Sir Salimullah Medical College, Mitford Hospital, Dhaka, Bangladesh

ROS1 rearrangements are a unique molecular subset of non-small cell lung cancer (NSCLC), seen in approximately 2% of non-squamous NSCLC and frequently affecting younger people and never-smokers^[^1^]^. Central nervous system (CNS) involvement is an important clinical challenge, and acquired ROS1 kinase-domain mutations, especially the solvent-front G2032R mutation, may lead to resistance to previous tyrosine kinase inhibitors (TKIs)^[^1,2^]^. While newer ROS1 inhibitors have yielded improved systemic and intracranial disease control, neurologic toxicity from off-target tropomyosin receptor kinase (TRK) inhibition remains a therapeutic consideration, and thus, it is important to have agents that are highly selective and brain-penetrable ^[^2,3^]^.

Zidesamtinib (NVL-520; Jideytro) is a next-generation, ROS1-selective TKI that seeks to overcome these limitations. The U.S. Food and Drug Administration granted approval to zidesamtinib for the treatment of adults with ROS1-positive, locally advanced or metastatic NSCLC who have previously received at least one ROS1 TKI^[^4^]^. Preclinical studies revealed ROS1 selectivity and low TRK activity, brain penetration, and activity against a wide range of ROS1-resistant mutations, such as G2032R^[^2,3^]^. Zidesamtinib induced long-lasting antitumor effects in an intracranial G2032R xenograft model, suggesting the potential for clinical evaluation in patients with CNS disease and acquired resistance^[^2^]^.

Clinical data come mainly from the multicenter, open-label, single-arm ARROS-1 trial (NCT05118789)^[^4,5^]^. The confirmed overall response rate (ORR) in the efficacy arm was 44% (95% CI, 34–53) in 117 patients with ROS1-positive NSCLC who had received prior treatment^[^5^]^. Of those responding, 82% maintained a response for ≥6 months and 69% for ≥12 months^[^5^]^. Responses were seen after one prior ROS1 TKI (49%) and after two or more prior ROS1 TKIs (38%), consistent with activity in heavily pretreated disease^[^4,5^]^. The response rate in the CNS was 48% (including complete responses in 22%) among 50 patients with measurable CNS metastases at baseline who had no prior brain radiotherapy within 2 months of study entry^[^5^]^. Activity was also seen against resistance mutations, with 14/26 patients (54%) harboring ROS1 G2032R^[^5^]^.

The safety profile appears reasonable but should be monitored. Common adverse effects are peripheral edema, peripheral neuropathy, constipation, tiredness, and breathlessness^[^5^]^. The clinically relevant warnings are CNS adverse reactions, prolongation of the QTc interval, interstitial lung disease/pneumonitis, skeletal fractures, myalgia, elevation of creatine phosphokinase (CPK), and embryo-fetal toxicity^[^4,5^]^. A total of 25% of patients had adverse reactions that were considered CNS adverse reactions; grade 3–4 events occurred in 2.5% of patients^[^5^]^. Therefore, ROS1 selectivity and TRK sparing do not mean there are no neurologic toxicities. Neurologic symptoms, QTc prolongation and electrolyte abnormalities, pulmonary toxicity, CPK elevation, and abnormalities of pancreatic enzymes should be assessed as part of clinical monitoring according to the prescribing information^[^5^]^.

The promising results, however, need to be taken with a grain of salt. The ARROS-1 single-arm study does not have a randomized comparator arm, and analyses stratified by CNS involvement and by mutation status are relatively small. In view of this, the information presented to date shows clinically relevant activity but does not demonstrate superiority to other next-generation ROS1 TKIs. However, results are also influenced by whether published or regulatory data cuts are used for the ARROS-1 data, so the reported results with respect to responses and intracranial outcomes should be interpreted with this in mind. Further follow-up and comparative studies are required to establish the optimal treatment sequence, to assess the use of zidesamtinib in first-line therapy, to define mechanisms of acquired resistance, and to determine long-term intracranial control.

Zidesamtinib will therefore be a clinically meaningful new option for ROS1-positive, previously treated NSCLC, especially in those with CNS metastases or G2032R resistance. Its ROS1 selectivity, TRK-sparing profile, activity against resistance mutations, and clinically proven CNS activity are compelling therapeutic reasons. However, the evidence that is available is largely nonrandomized, and comparative efficacy has not yet been demonstrated. Future prospective comparative studies will help to determine its best place in the developing landscape of ROS1-targeted therapy.

## Data Availability

Available to S.B.I.
